# Novel Environmentally Responsive Polyvinyl Polyamine Hydrogels Capable of Phase Transformation with Temperature for Applications in Reservoir Profile Control

**DOI:** 10.3390/gels9120950

**Published:** 2023-12-04

**Authors:** Jianxun Meng, Guoliang Mao, Zhixuan Zhu, Qingsong Li, Xuesong Lin, Lichao Wang, Yiran Li, Yue Huang

**Affiliations:** 1 College of Chemistry and Chemical Engineering, Northeast Petroleum University, Daqing 163318, China; mengjianxun@petrochina.com.cn; 2 Research Institute of Oil Production Engineering, Daqing Oilfield Limited Company, Daqing 163453, China; liqingsong@petrochina.com.cn (Q.L.); linxuesong@petrochina.com.cn (X.L.); wanglichao_cyy@petrochina.com.cn (L.W.); liyiran@petrochina.com.cn (Y.L.); 3 Heilongjiang Provincial Key Laboratory of Oil and Gas Reservoir Stimulation, Daqing 163453, China; 4 No. 2 Production Plant, Daqing Oilfield Limited Company, Daqing 163461, China; haungyezi@163.com

**Keywords:** temperature-sensitive hydrogel, environmentally response, phase transformation, profile control performance, enhanced oil recovery

## Abstract

Hydrogel has been widely used in reservoir regulation for enhancing oil recovery, however, this process can experience negative influences on the properties and effects of the hydrogels. Therefore, developing novel hydrogels with excellent environmental responsiveness would improve the formation adaptability of hydrogels. In this study, novel polyvinyl polyamine hydrogels were synthesized by a ring-opening addition reaction between polyvinyl polyamines and polyethylene glycol glycidyl ether. The results of atomic force microscopy and transmission electron microscopy showed that the polyvinyl polyamine gel had a porous and irregular bulk structure and was endowed with water storage. With the temperature rising from 30 °C to 60 °C, the transmittance of diethylenetriamine hydrogel decreased from 84.3% to 18.8%, indicating that a phase transition had occurred. After the polyvinyl polyamine hydrogel with low initial viscosity was injected into the formation in the liquid phase, the increase of the reservoir temperature caused it to turn into an elastomer, thereby migrating to the depth of the reservoir and achieving effective plugging. Polyvinyl polyamine hydrogel could improve the profile of heterogeneous layers significantly by forcing subsequent fluids into the low permeability zone in the form of elastomers in the medium temperature reservoirs of 40–60 °C. The novel environmentally responsive polyvinyl polyamine hydrogels, capable of phase transformation with temperature, exhibited superior performance in recovering residual oil, which was beneficial for applications in reservoir profile control and oilfield development.

## 1. Introduction

Hydrogels, with water as a dispersing medium, can swell rapidly in water and retain a large volume of water without dissolving in this swelled state [1]. The hydrophilicity of hydrogels is mainly due to the hydrophilic groups on the main chain, while the three-dimensional network crosslinking structure ensures that the hydrogel is not dissolved in water [2,3,4,5]. Therefore, as a non-toxic material with excellent viscoelasticity, hydrogel has been widely used in the petrochemical industry [6,7]. Heterogeneity of the reservoir makes profile control a necessary means to increase crude oil production. When the hydrogel with polymers and crosslinkers as main components is injected into the reservoir, it preferentially plugs the high permeability area to control the profile of the heterogeneous reservoir, so that the subsequent injected fluids can sweep the crude oil into the low permeability area [8]. However, the profile control effect of conventional hydrogels is probably weakened due to ion adsorption and shear dilution within the formation [9]. With the increasingly harsh reservoir environment, the demand for hydrogels has also increased, and smart hydrogels with environmental responsiveness have emerged [10]. When the smart hydrogel is subjected to small physical and chemical stimulation, its properties can be significantly changed.

Temperature is one of the most important environmental factors affecting the application effect of smart hydrogels. Since the temperature-sensitive polyisopropylacrylamide (PNIPAM) hydrogel was synthesized for the first time in 1978, it has attracted lots of attention [11]. A temperature responsive hydrogel containing PNIPAM induced by dual supramolecular assemblies appeared as a liquid dispersed phase when the temperature was lower than the critical temperature [12]. When the temperature rises above the critical temperature, The supramolecular hydrogel transforms into a solid elastomer. With the action of the simple PNIPAM based copolymers, a photoacid could be used to capture a chemical signal when the light varied [13]. The lower critical solution temperature of the PNIPAM copolymers was changed to promote the development of a signal transformation strategy. The nanoparticles were covalently grafted onto poly(N-isopropyl acrylamide) (PNIPAM) chains and then entangled with the polymer matrix of the membrane [14]. Membranes with PNIPAM graft fillers do not form pinholes at the interface between filler and the matrix, making the membrane technology potentially superior in terms of filtration performance. The thermosensitive poly(N-isopropylacrylamide) could be also grafted with magnetic-cored dendrimers to make the internal cavities of lipophilic PNIPAM-g-MCD adsorb benzene, resulting in a thermodynamically stable state and an increase in the removal efficiency of benzene [15]. Gelatin and N-isopropylacrylamide can be used as raw materials for preparing microbial resistant thermosensitive Ag nanocomposite hydrogels, which have exhibited very strong antibacterial activities [16]. The chitosan-based thermosensitive hydrogel containing chitosan, hydroxypropyl methylcellulose and glycerol was developed by Wang et al. [17]. The large amount of hydrophobic interactions in hydroxypropyl methylcellulose are conducive to thermal gelation of this hydrogel. The excellent fluidity and biodegradability has ensured that this hydrogel can be used in the field of biomedicine. Hydrogels loaded with glucosamine can undergo a sol–gel transition reaction at 35 °C, allowing glucosamine to be slowly released from the heat-sensitive hydrogel [18]. The swelling of the hydrogel group was reduced, while the application of thermosensitive hydrogels in the effective treatment of osteoarthritis was realized. By end-grafting the multiblock copolymer of water-soluble polymers, polyethylene glycol (PEG), a temperature-responsive hydrogel with biocompatibility, can be obtained [19]. The phase transition temperature of the hydrogel can be adjusted by changing the molecular weight of the block polymer. Nevertheless, the aforementioned hydrogels are mainly used in the biomedical field and do not have good adaptability for applications in reservoir profile control. A temperature-responsive hydrogel (PFAB), synthesized by combining a dynamic covalent cross-linked network with a temperature-resistant polymer network, can achieve a shunt rate of 80% in heterogeneous reservoirs at 120 °C, which can effectively profile the control profile to enhance oil recovery [20,21]. However, PFAB is only suitable for high-temperature reservoirs on account of substandard strength at medium temperature. Medium temperature formations of 40–60 °C are still being developed in the main reservoir at present; however, little study has been conducted on environmental response hydrogels for medium temperature reservoirs. The profile control effect of conventional hydrogels on the medium temperature reservoir is limited due to injection factors such as the chromatographic separation effect, and hence, it is of great significance to develop smart hydrogels that are only sensitive to temperature.

Herein, a series of temperature-sensitive polyvinyl polyamine hydrogel agents with adjustable gel-forming temperature in the range of 40–60 °C were obtained by using ethylene glycol glycidyl ether solution and polyvinyl polyamine to react. The structure was characterized by atomic force microscopy and transmission electron microscopy. The compressive strength of the hydrogel elastomer after phase transformation was measured and the profile control performances of the hydrogel were obtained through core tests. The development of this temperature-sensitive hydrogel has a guiding significance for improving the adaptability of hydrogels to the environmental reservoir to enhance oil recovery, and provides support for the construction of materials with environmental response characteristics.

## 2. Results and Discussion

### 2.1. Characterizations of the Synthesized Hydrogels

**Figure 1:**
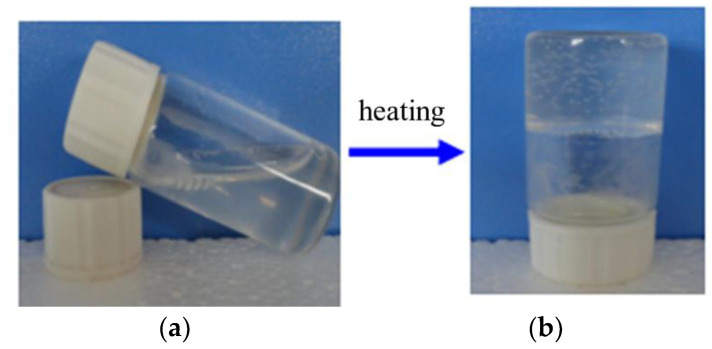
States of a representative synthesized product: (**a**) 25 °C; (**b**) 45 °C.

The states of a representative synthesized product at different temperatures are shown in Figure 1. The molecular chains of the synthesized hydrogel were dispersed at 25 °C. The storage modulus of the synthesized diethylenetriamine hydrogel was 0.73 Pa and the product could be seen as liquid (Figure 1a). As shown in Figure 1b, when the experimental environment temperature rose to 45 °C, the product presented a state of elastomer with a storage modulus of 983.11 Pa. When taking diethylenetriamine hydrogel as the example, the average storage moduli of this system was 0.73 Pa at 25 °C and 983.11 Pa at 45 °C, which was caused by the phase transformation. The rise in temperature caused the water molecules to be more amenable to interact with themselves, repelling hydrophobic groups. The aggregation of hydrophobic groups enhanced the interaction between polymer chains, leading to a denser network structure of the synthesized hydrogel. The presence of elastomers represented a phase transformation with temperature, indicating that the polyvinyl polyamine hydrogels exhibited a temperature dependence.

**Figure 2:**
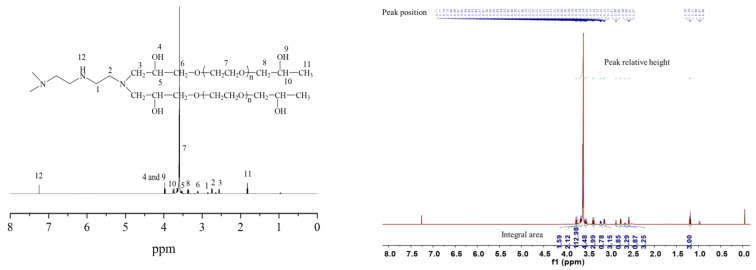
Nuclear magnetic resonance hydrogen spectra of hydrogels bridged by polyvinyl polyamine and PEGO when bridging group was diethylenetriamine.

It was seen from the nuclear magnetic resonance hydrogen spectra in Figure 2 that the synthesis reaction between diethylenetriamine and PEGO took place successfully. As demonstrated in Figure 2 the characteristic peak of the hydrogen proton on the methylene connected with the secondary amine appeared at a chemical shift of 2.85 ppm, while the characteristic peak of the hydrogen proton on the methylene connected with the tertiary amine appeared at chemical shifts of 2.76 ppm and 2.56 ppm, respectively. The characteristic peak of the hydrogen proton on the hydroxyl group appeared at a chemical shift of 3.98 ppm. The characteristic absorption peak of the hydrogen proton on the methylene connected with the oxygen atom in the molecular chain of polyethylene glycol glycyl ether appeared at a chemical shift of 3.13 ppm. The characteristic peak of the hydrogen proton on the methylene on oxyethyl appeared at a chemical shift of 3.59 ppm, while the characteristic peak of the hydrogen proton on the methylene connected to the oxyethyl appeared at a chemical shift of 3.36 ppm. The characteristic peaks of the hydrogen proton on the submethyl group connected to the hydroxyl group appeared at 3.53 ppm and 3.72 ppm, respectively, and the characteristic peak of the hydrogen proton on the methyl group appeared at a chemical shift of 1.81 ppm. The presence of the above characteristic peaks of hydrogen protons indicated that diethylenetriamine and PEGO had undergone a ring opening addition reaction [22].

**Figure 3:**
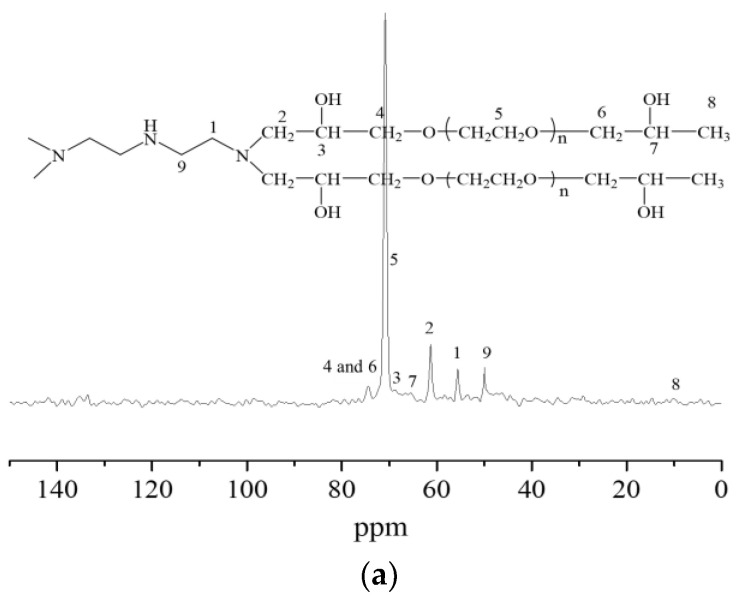

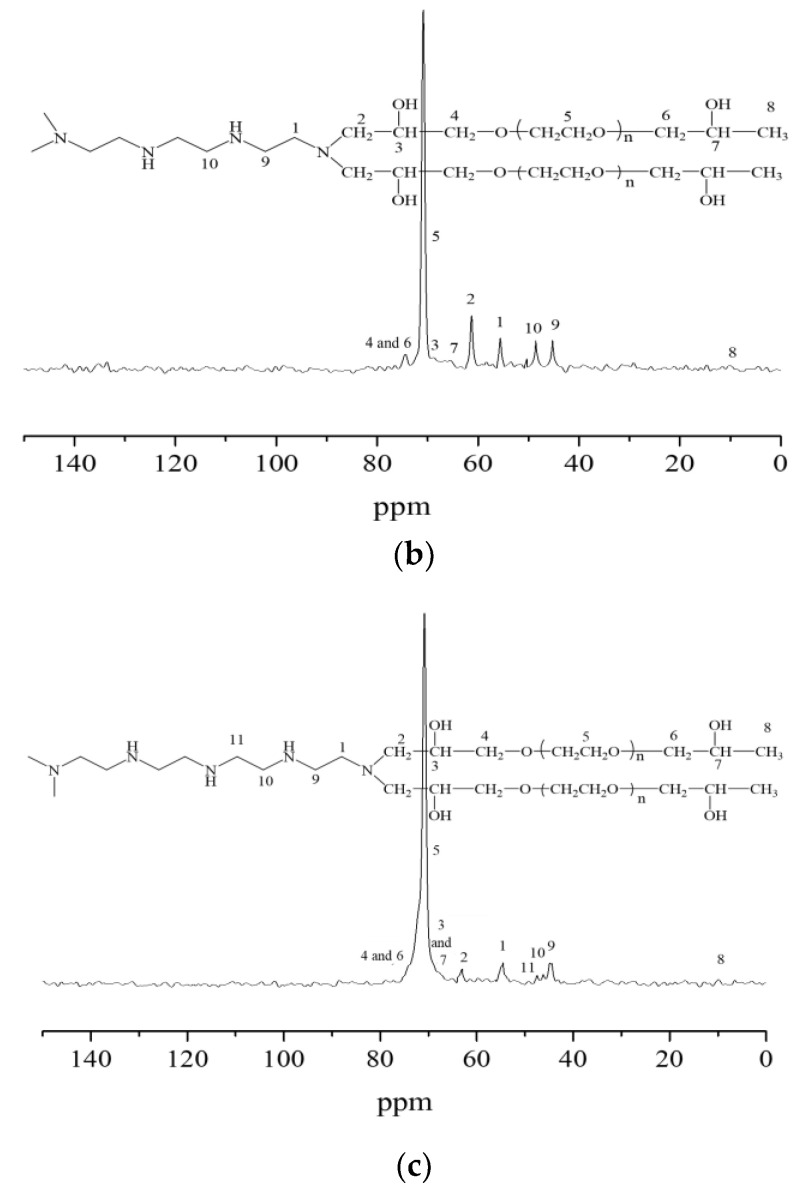
Nuclear magnetic resonance carbon spectra of hydrogels bridged by polyvinyl polyamine and PEGO. (**a**) Bridging group was diethylenetriamine; (**b**) bridging group was triethylene tetramine; (**c**) bridging group was tetraethylenepentamine.

The nuclear magnetic resonance carbon spectra of the synthesized hydrogels are described in Figure 3. When taking Figure 3c as an example, it was seen that the characteristic peaks of carbon atoms in the methylene chain connected with secondary amine appeared at chemical shifts of 44.66 ppm, 26.18 ppm, and 47.59 ppm, respectively. The characteristic peaks of the carbon atoms in the methylene chain connected to the tertiary amine appeared at a chemical shift of 54.65 ppm. The characteristic peaks of carbon atoms in the methylene group connected to the tertiary amine in the molecular chain of polyethylene glycol glycidyl ether appeared at a chemical shift of 63.16 ppm. The characteristic peaks of carbon atoms in the submethyl group connected with the hydroxyl group appeared at a chemical shift of 67.09 ppm. At the chemical shift of 74.08 ppm, the characteristic peaks appeared of carbon atoms in the methylene-connected methylene group in the molecular chain of PEGO ether. The characteristic peaks of carbon atoms in the ethoxy group appeared at a chemical shift of 70.70 ppm, while the characteristic peaks of carbon atoms in the end group methyl group appeared at a chemical shift of 9.96 ppm. The presence of the above characteristic peaks of carbon atoms further indicated that the ring-opening addition reaction had occurred between tetraethylene pentamine and PEGO, proving the successful synthesis of the hydrogels.

**Figure 4:**
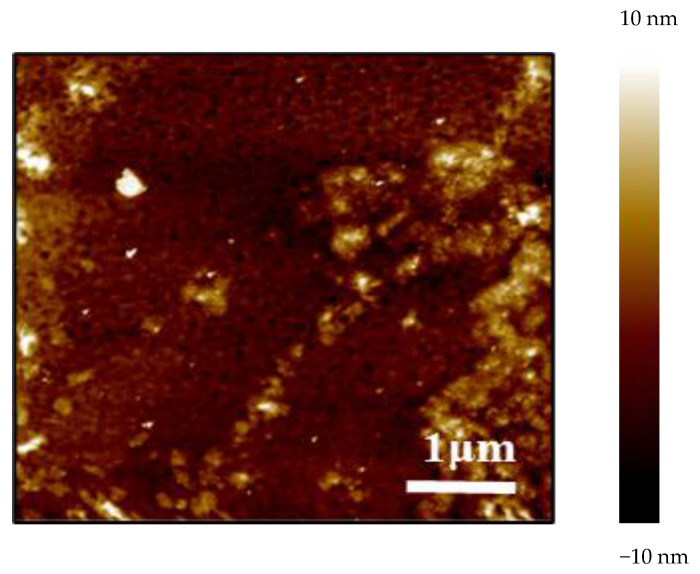
Microscopic morphology of diethylenetriamine polyvinyl polyamine hydrogel.

The micro-topographies and properties of the surface of the three synthesized polyvinyl polyamine hydrogels were similar to each other. A representative AFM result is shown in Figure 4. The molecular chains exhibited a state of interweaving, aggregation, and entanglement. The surface of the synthesized hydrogel was very rough with a pinhole structure, presenting a three-dimensional spatial network structure as a whole [23]. This unique structure could provide sufficient storage space for adsorbing water, so that the polyethylene polyamine hydrogel could deform and adjust the profile of the reservoir [24,25].

**Figure 5:**
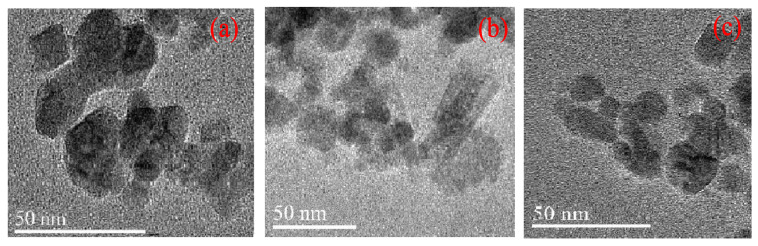
TEM results of polyvinyl polyamine hydrogel. (**a**) Diethylenetriamine hydrogel; (**b**) triethylene tetramine hydrogel; (**c**) tetraethylenepentamine hydrogel.

As illustrated in Figure 5, all the synthesized polyvinyl polyamine hydrogels had similar morphologies, showing obvious irregular block structures. Each single large structure is made up of many small granular structures of different shapes, and the edge of some of the small particle samples can be distinguished with an obvious angular structure [26]. There were porous pores on the surface of the particles, which is beneficial for the hydrogel to store water [27].

### 2.2. Effect of Temperature on Phase Transformation and Environmental Responsiveness

**Figure 6:**
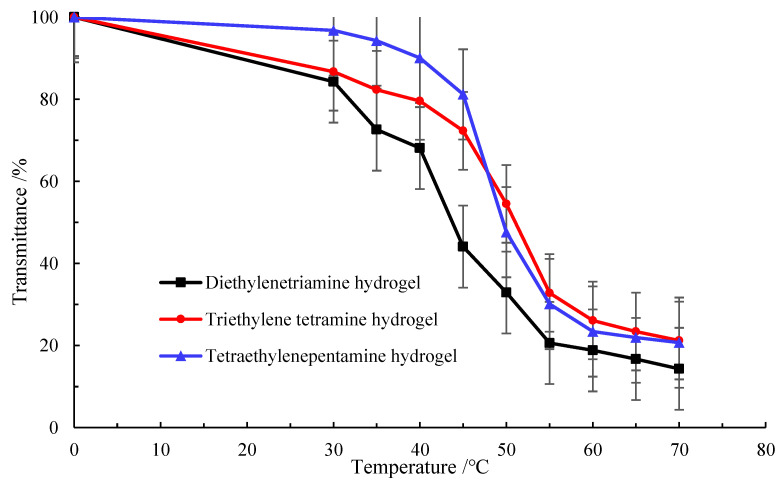
Variation curves of polyvinyl polyamine hydrogel with temperature.

It is seen from Figure 6 that when the test temperature gradually increased to 40 °C, the light transmittance of the three polyvinyl polyamine hydrogels decreased significantly. As a result, the temperature of 40 °C could be seen as the critical temperature, at which the systems began to undergo phase transformation and gradually became elastomeric. When the temperature rose to above 60 °C, the transmittance still decreased but only slightly. This was explained in that the synthesized environmentally responsive polyvinyl polyamine hydrogel contained a large number of hydrophilic groups and hydrophobic groups, and when the temperature changed, the interaction between these groups and water led to the phase transformation of the hydrogel. When the temperature was lower than the phase transformation temperature, the hydrophilic groups and water molecules were combined with each other in the form of hydrogen bonds. The hydrogel swelled due to the absorption of water, resulting in the dispersion of the molecular chain [28,29]. As a result, the temperature of 40 °C could be seen as the critical temperature, at which the systems began to undergo phase transformation and gradually became elastomeric. Polyvinyl polyamine is relatively cheap and suitable for industrial production. The higher the concentration of PEGO-600, the shorter the gelation time of the hydrogel. In addition, the higher the hydrogel concentration, the lower is the critical phase transition temperature. PEGO acts as a response group [30]. Macromolecules containing ether bonds and amine roots enhance the temperature response sensitivity of hydrogels [31]. The synthesized hydrogels could respond to changes in temperature. At lower temperatures, polymer chains might be in a looser state due to intermolecular hydrogen bonds and hydrophobic interactions. As the temperature rises to between 40 °C and 60 °C, hydrophobic forces made the hydrophobic groups, such as the long-chain hydrocarbons in hydrogels, close to each other and aggregate under the action of thermal motion. The interactions between polymer chains are enhanced, resulting in a more ordered polymer network structure. This change in structure causes the hydrogel to change from a loose state to a more compact and elastic state; the hydrogels present the state of elastomer. The hydrogel is in this state at 40–60 °C, and hence, it can better maintain its shape and structure and return to the original state even after applying external forces.

Table 1 illustrated the effect of temperature on the plugging rate of the representative tetraethylenepentamine hydrogel on the sand-packed pipe. The produced liquid volume was almost the same as the injected pore volume at 20–30 °C, indicating that the liquid phase hydrogel had low initial viscosity and excellent injection performance. However, it was difficult for the polyvinyl polyamine hydrogels before phase transformation to plug the simulation core effectively.

With the increase of ambient temperature, the phase transformation of hydrogels was caused by the effect of both the physical action (including charge interaction, hydrophobic interaction, and hydrogen bonding) and the chemical action of dynamic covalent bonds [32]. The hydration capacity of the polyvinyl polyamine hydrogels could be regulated by raising the temperature, while the flow resistance in the pore throat structure increased with the rise of the temperature [33]. When the experimental temperature was between 40 and 60 °C, the elastic deformation of the hydrogel resulted in a significant increase in the plugging rate. Some hydrogels were adsorbed and retained in the relatively large pore throats, which reduced the permeability of the simulation core, resulting in a plugging rate of more than 85%. Excessive temperature might cause the structure of part of the hydrogel to be destroyed [34]. Consequently, the deduction that polyvinyl polyamine hydrogels have a good ability to regulate the 40–60 °C medium temperature reservoir was concluded.

### 2.3. Profile Improvement Mechanism of Polyvinyl Polyamine Hydrogels

**Figure 7:**
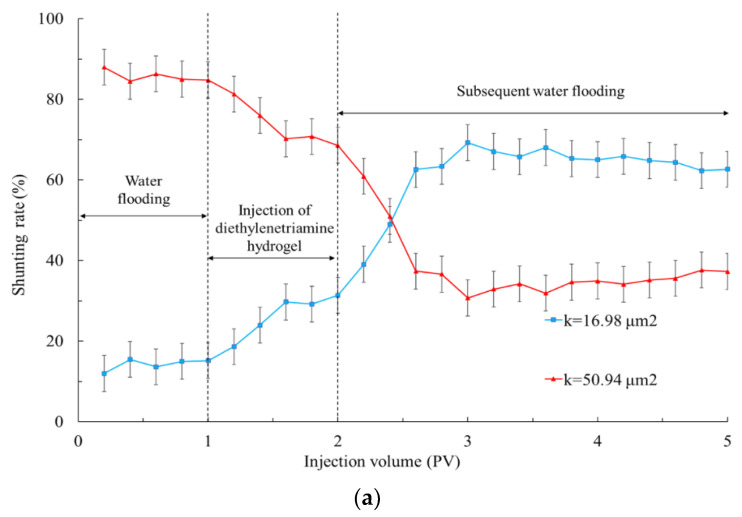

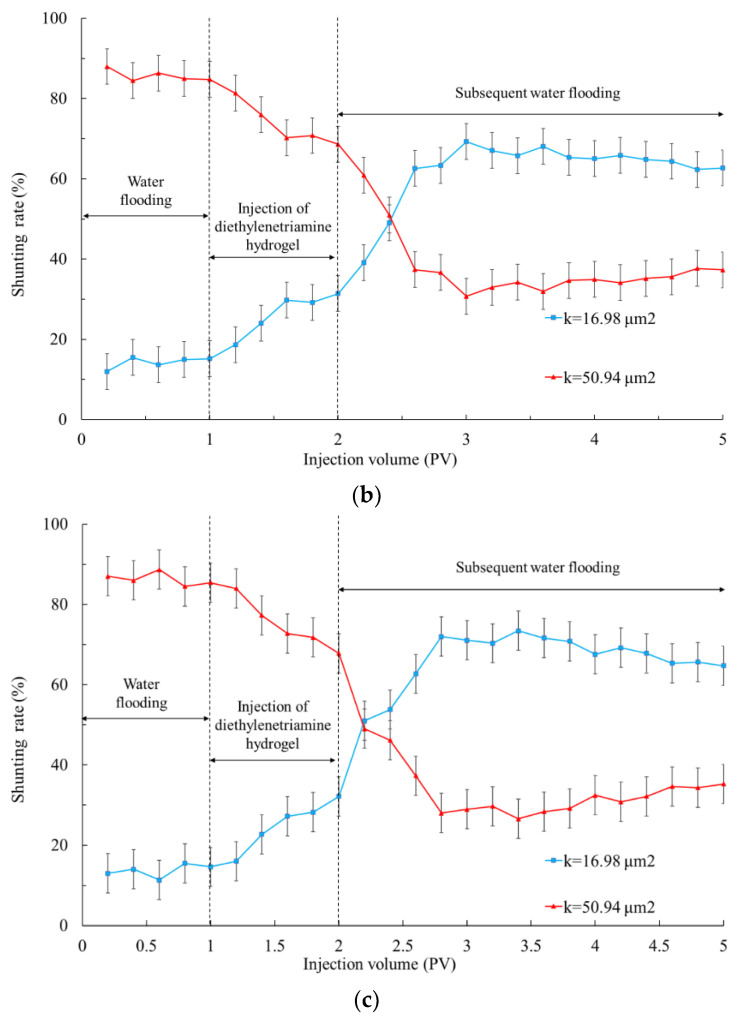
Shunting rate when injecting polyvinyl polyamine hydrogels into double-layer simulation cores at 40 °C. (**a**) Diethylenetriamine hydrogel; (**b**) triethylene tetramine hydrogel; (**c**) tetraethylenepentamine hydrogel.

It can be seen from Figure 7 and Table 2 that when polyvinyl polyamine hydrogels were injected into heterogeneous layers, the liquid production of the low permeability tube increased significantly. These phenomena were due to the fact that when the polyvinyl alcohol polyamine hydrogel was first injected, it appeared as liquid phase [35]. With the continuous migration of hydrogels to the depth of the formation, the increase in temperature caused the hydrogel to undergo a phase transformation into an elastomer, which was equivalent to delaying cross-linking. The hydrogel with low initial viscosity was not affected by ion adsorption and shear dilution during injection, and was only sensitive to temperature, so that the hydrogels selectively reduced the permeability of the heterogeneous porous media [36]. The plugging effects of the hydrogels that became elastomers after the phase transformation at 40 °C on the high permeability layer made the fluid enter the low permeability area during the subsequent water flooding. Therefore, the shunting ability of the low permeability layer became gradually superior to that of the high permeability zone [37].

The three polyvinyl polyamine hydrogel systems, whose phase transformation interval with temperature was 40–60 °C, had a certain elasticity. The injection of environmentally responsive hydrogel was conducive to the adjustment of the seepage profile and the expansion of sweep efficiency. The physical barrier effect was formed by the plugging of elastic hydrogel in the near well area to adjust the homogeneity of the formation profile and the relationship between oil and water production, so as to reduce the water cut of the produced liquid [38]. The subsequent injection of fluid could maximize the effect on the low permeability layer and the area containing low oil saturation, thereby playing the dual role of water control and oil displacement. Due to the easy access to monomer raw materials and low cost, using polyvinyl polyamine hydrogel as a temperature-sensitive profile control agent could achieve the purpose of enhancing crude oil recovery of medium temperature reservoirs.

## 3. Conclusions

In this study, hydrophilic groups that can interact with water were introduced into hydrogels from the perspective of the temperature sensitive and environmentally responsive profile control agent. The transmittance of tetraethylenepentamine hydrogel was 90.1% at 40 °C, and it gradually decreased to 31.2% as the temperature rose to 60 °C. The synthesized three polyvinyl polyamine hydrogels presented a liquid phase below 40 °C and a solid phase at 40–60 °C due to the interaction of hydrophobic groups. The dense network structure ensured the polyvinyl polyamine hydrogel had excellent water storage capacity, and the low initial viscosity ensured migration to the target layer, thus effectively improving the heterogeneity of profiles in the form of elastomers. The plugging rate of tetraethylenepentamine hydrogel to the reservoir with a permeability of 16.98 μm^2^ was 87.81%. The profile improvement rate of polyvinyl polyamine hydrogels for the 40–60 °C reservoir can achieve more than 91.88%. By adjusting the ratio of PEGO and polyvinyl polyamine ratio, the temperature response interval of the hydrogel changes accordingly, and the phase transformation causes the subsequent injection fluid to sweep for the displacement of crude oil in the low permeability zone. The single-component polyvinyl polyamine hydrogel developed in this paper, which is controlled only by formation environmental factors, shows great potential in profile controlling and can be used as the rear slug when injecting the traditional plugging agent. The development of an environmentally responsive agent has very broad prospects for the sustainable exploitation of oilfields, providing guidance for designing smart materials for specific applications.

## 4. Materials and Methods

### 4.1. Materials

Polyethylene glycol glycidyl ether (PEGO) was synthesized using polyethylene glycol, epichlorohydrin (ECH) with the relative molecular mass of 92.52 g/mol, tetrabutylammonium bromide (TBAB) with the relative molecular mass of 322.37 g/mol and sodium hydroxide (NaOH) purchased from Shanghai Maclin Biochemical Co., Ltd. (Shanghai, China). The purities of these four chemicals were all analytically pure for the preparation of PEGO-600. The bridging group polyvinyl polyamine grafted on PEGO-600 (including diethylenetriamine, triethylenetetramine, and tetraethylenepentamine) was purchased from Aladdin Chemical Reagent Co., Ltd. (Shanghai, China).

### 4.2. Synthesis of Polyvinyl Polyamine Hydrogels

**Figure 8:**
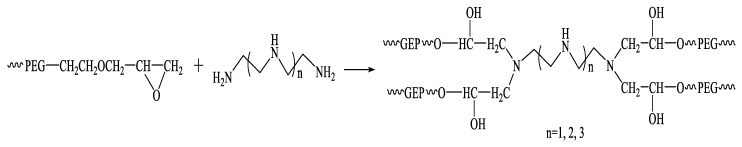
Synthetic route of hydrogel bridged by polyvinyl polyamine and PEGO.

As shown in Figure 8, the polyvinyl polyamine hydrogel was synthesized from polyvinyl polyamine and polyethylene glycol glycidyl ether by a ring opening addition reaction. Then 0.3% PEG and 0.2% ECH were added to ultra-pure water and stirred until the solution was uniform and 0.05% TBAB was added to play the role of phase transfer. After the stirring speed was increased to 800 RPM, 0.06% NAOH was added to the solution containing the three materials, and the reaction was carried out at 40–60 °C for 6 h to obtain the polyethylene glycol glycidyl ether (PEGO).

The filtered and distilled PEGO was prepared in a 17.5% solution. The solution was reacted with 0.4–1.7% polyvinyl polyamines at different response temperatures ranging from 40 °C to 60 °C for 16–72 h to prepare the hydrogel bridged by polyvinyl polyamines and PEGO. The above experiments were all carried out under a condition of environmental pH equal to 7.

### 4.3. Structural Characterization of Polyvinyl Polyamine Hydrogel

Rheology tests were carried at 25 °C and 45 °C to measure the storage moduli of the polyvinyl polyamine hydrogel using a rheometer. Before the storage moduli of hydrogels were obtained, the linear viscoelastic region was determined by stress scanning in the oscillatory shear mode and the angular frequency was set as 6.28 rad/s. Nuclear magnetic resonance spectrometry (NMR spectrometer, JNM-ECZ500R) manufactured by Japanese electronics company (Tokyo, Japan) was used to measure the hydrogen spectra and carbon spectra of the polyvinyl polyamine hydrogel. Atomic force microscopy (AFM, XE-7) manufactured by American Park company (Frisco, TX, USA) and transmission electron microscopy (TEM, TecnaiG2 F20 S-TWIN) manufactured by American FEI company (Hillsboro, OR, USA) were used to analyze the microstructure of the synthesized hydrogel. 

### 4.4. Temperature Sensitivity Test of Polyvinyl Polyamine Hydrogel

Ultraviolet tests and plugging capacity were used to evaluate the temperature response performance of the polyvinyl polyamine hydrogel. The synthesized hydrogel was placed in a UV-visible spectrophotometer with a constant temperature water bath after swelling fully. The transmittance experiment was conducted under the condition whereby the wavelength was adjusted to 560 nm with a heating rate of 0.5 °C/min. The change of light transmittance (T) of the sample with temperature in the range of 40 °C to 60 °C was recorded at an optical path of 3 cm [39].

The plugging rates of the synthesized polyvinyl polyamine hydrogel before and after phase transformation were measured by a flooding experiment in a Φ25 mm × 200 mm single sand-packed pipe with porosity of about 40%. The initial permeability *k*_wi_ of the experimental core model was 16.98 μm^2^, which simulated the permeability of the formation of the Daqing oilfield western Sapu block. The simulation core was first placed in an oven of 30 °C, and the synthesized hydrogels were injected into the simulation core at a speed of 0.5 mL/min until the injection volume reached 1 pore volume (PV). Then the temperature of the simulation core was heated to 40–70 °C. The core permeability injected with 1 PV hydrogel at different temperatures was recorded as *k*_wt_. The plugging capacity of polyvinyl polyamine hydrogel was obtained using the following equation:

*P* = (*k*_wt_ − *k*_wi_)/*k*_wi_ × 100% (1)

where *P* was the plugging rate.

### 4.5. Profile Control Performance of Polyvinyl Polyamine Hydrogel

**Figure 9:**
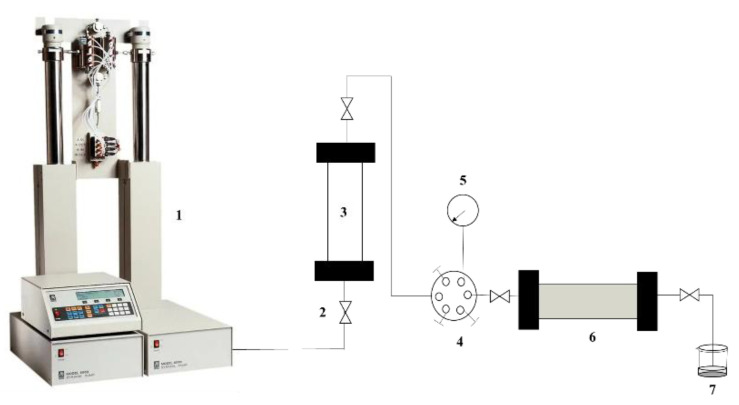
Schematic of the physical simulation double-layer cores device: 1. ISCO pump, 2. valve, 3. intermediate container with polyvinyl polyamine hydrogel, 4. six-way valve, 5. pressure sensor, 6. sand-packed pipe, 7. beaker collected with produced fluid.

The profile control experiment was carried out in the simulation double-layer cores with similar permeability contrast of 1:3, as depicted in Figure 9. Two Φ25 mm × 200 mm simulation cores were placed in parallel to simulate double-layer heterogeneous formation. The initial permeabilities of the high and low-permeability sand-packed pipes were 50.94 μm^2^ and 16.98 μm^2^ respectively. The water absorption rates of the high and low-permeability pipes before profile control were *Q*_lb_ and *Q*_hb_ respectively. Polyvinyl polyamine hydrogels were injected into the double-layer cores at a constant injection rate of 0.5 mL/min.

The double-layer pipes injected with polyvinyl polyamine hydrogels were aged at 40 °C for 30 days. Then the subsequent water was injected into the double-layer stimulation core until the pressure at the output end became stable. The water absorption rates of the high and low-permeability pipes during subsequent water flooding were *Q*_la_ and *Q*_ha,_ respectively. The shunting rate *f*, which characterized the profile control performance of the polyvinyl polyamine hydrogel, could be calculated using the following equation:

(2) $$f=(1-\frac{Q_{\mathrm{ha}}}{Q_{\mathrm{la}}}/\frac{Q_{\mathrm{hb}}}{Q_{\mathrm{lb}}})\times 100\%$$

## Figures and Tables

**Table 1:** Plugging performances of hydrogel at different temperatures.

System	Temperature/°C	*k*_wi_/ μm^2^	*k*_wt_/ μm^2^	Plugging Rates/%	Standard Deviation/%
Tetraethylenepentamine hydrogel	20	16.98	14.11	16.8	2.7
	30		13.79	18.79	3.9
	40		2.24	87.81	3.1
	50		1.98	88.34	1.8
	60		2.09	87.69	5.2
	70		9.37	44.82	4.5

**Table 2:** The profile improvement capacity of polyvinyl polyamine systems.

System	Core Type	Initial Permeability	Shunting Rate (%)		Profile Improvement Rate (%)
			Before Injecting Hydrogel	After Injecting Hydrogel	
Diethylenetriamine hydrogel	Low permeability	16.98	12	62.68	91.88
	High permeability	50.94	88	37.32	
Triethylenetetramine hydrogel	Low permeability	16.98	13	71.24	93.97
	High permeability	50.94	87	28.76	
Tetraethylenepentamine hydrogel	Low permeability	16.98	13	64.72	91.86
	High permeability	50.94	87	35.28	

## Data Availability

All data and materials are available on request from the corresponding author. The data are not publicly available due to ongoing research using a part of the data.
